# Genome-Wide Mapping of Virulence in Brown Planthopper Identifies Loci That Break Down Host Plant Resistance

**DOI:** 10.1371/journal.pone.0098911

**Published:** 2014-06-09

**Authors:** Shengli Jing, Lei Zhang, Yinhua Ma, Bingfang Liu, Yan Zhao, Hangjin Yu, Xi Zhou, Rui Qin, Lili Zhu, Guangcun He

**Affiliations:** 1 State Key Laboratory of Hybrid Rice, College of Life Science, Wuhan University, Wuhan, China; 2 Engineering Research Center of Protection and Utilization for Biological Resources in Minority Regions, College of Life Science, South-Central University for Nationalities, Wuhan, China; National Key Laboratory of Crop Genetic Improvement, China

## Abstract

Insects and plants have coexisted for over 350 million years and their interactions have affected ecosystems and agricultural practices worldwide. Variation in herbivorous insects' virulence to circumvent host resistance has been extensively documented. However, despite decades of investigation, the genetic foundations of virulence are currently unknown. The brown planthopper (*Nilaparvata lugens*) is the most destructive rice (*Oryza sativa*) pest in the world. The identification of the resistance gene *Bph1* and its introduction in commercial rice varieties prompted the emergence of a new virulent brown planthopper biotype that was able to break the resistance conferred by *Bph1.* In this study, we aimed to construct a high density linkage map for the brown planthopper and identify the loci responsible for its virulence in order to determine their genetic architecture. Based on genotyping data for hundreds of molecular markers in three mapping populations, we constructed the most comprehensive linkage map available for this species, covering 96.6% of its genome. Fifteen chromosomes were anchored with 124 gene-specific markers. Using genome-wide scanning and interval mapping, the *Qhp7* locus that governs preference for *Bph1* plants was mapped to a 0.1 cM region of chromosome 7. In addition, two major QTLs that govern the rate of insect growth on resistant rice plants were identified on chromosomes 5 (*Qgr5*) and 14 (*Qgr14*). This is the first study to successfully locate virulence in the genome of this important agricultural insect by marker-based genetic mapping. Our results show that the virulence which overcomes the resistance conferred by *Bph1* is controlled by a few major genes and that the components of virulence originate from independent genetic characters. The isolation of these loci will enable the elucidation of the molecular mechanisms underpinning the rice-brown planthopper interaction and facilitate the development of durable approaches for controlling this most destructive agricultural insect.

## Introduction

The coevolution of host plants and their enemies is a dynamic process that has spurred adaptation in both natural and agricultural systems [1]-[6]. This process is driven by reciprocal evolutionary interactions between the resistance of the host plant and the virulence of their enemies. Selective pressure prompts the host plant to evolve some new resistance character that reduces the damage caused by its enemy; in order to avoid extinction, the enemy must then evolve new virulence characters to overcome the plant's defenses or resistance. This process has occurred in the interactions of plants with both pathogens and insects, the latter of which have coexisted with plants for over 350 million years [7].

Many classic interactions between plants and herbivorous insects have been identified in agricultural systems, such as that between rice (*Oryza sativa* L.) and the brown planthopper (*Nilaparvata lugens* St ål). The brown planthopper is the most destructive insect rice pest and poses a serious threat to rice production in temperate and tropical Asia [8]. The variation in its ability to adapt to resistance in its host has been documented extensively in recent decades. The first virulence variation in brown planthopper was recorded in the 1970's, when rice varieties carrying the *Bph1* resistance gene were introduced commercially in order to control this pest. The selection pressure caused by this resistance led to the emergence of a new virulent planthopper population that tolerated the effects of *Bph1* [9], [10]. Virulence in brown planthoppers is defined as the ability to tolerate a given resistant rice variety or host plant resistance gene. This first virulent *N. lugens* population that had adapted to the resistance conferred by the *Bph1* gene was termed Biotype 2. In contrast, populations that can only survive on rice varieties which do not carry any brown planthopper resistance gene, such as the TN1 variety, are known as Biotype 1. With the release of more rice varieties carrying different resistance genes, a wider range of brown planthopper biotypes have been recorded [11]. These rice varieties carrying different resistance genes and the corresponding virulent brown planthopper populations thus provide an ideal system for studying the interactions between and coevolution of plants and herbivorous insects. Brown planthoppers aside, biotypes have been identified in insects such as aphids [12], whitefly [13], the hessian fly [14], gall midges [15] and the sweetpotato whitefly [16]. At present, little is known about the genetic architecture of virulence in herbivorous insects or the evolutionary interactions between plants and their insect pests.

While there is little data on systems involving plants and herbivorous insects, those involving plants and microbes have been studied extensively, leading to the development of a four phase ‘zigzag’ model [17], [18]. It is now understood that molecules known as pathogen-associated molecular patterns (PAMPs) and effectors play key roles in the virulence of pathogens towards plants. The first phase of plant resistance is the basal defense, in which the pathogen's PAMPs are recognized by the plant's pattern recognition receptors (PRR), initiating PAMP-triggered immunity (PTI). This prompted the evolution of virulence effectors in pathogens to suppress PTI. As a result, plants evolved *R* genes that mediate specific pathogen resistance mechanisms in which the *R* protein acts as a receptor that recognizes the pathogen's effectors, inducing effector-triggered immunity (ETI). Effector proteins are usually the products of virulence genes and can be regarded as virulence factors in pathogens [19] and aphids [20]. The relationship between the R and Avr genes is consistent with the gene-for-gene model [21]. All of these findings are likely to be relevant in studies on the interactions of plants and insect herbivores because plants may well use similar defensive strategies to cope with the effects of herbivore attacks [11].

Brown planthopper resistance was first reported in the Mudgo rice variety in 1969, and the first resistance gene, *Bph1*, was detected in the same variety in 1971 [22], [23]. A total of 28 different brown planthopper resistance genes have since been identified in cultivated and wild rice species, all of which are located on specific regions of the rice chromosomes [24], [25]. Of these resistant genes, only the *Bph14* gene has been isolated and characterized via a map-based cloning strategy [26]. The *Bph14* gene encodes a coiled-coil, nucleotide-binding, and leucine-rich repeat (CC–NB–LRR) protein of the NB–LRR family that resembles the R proteins that contribute to plant resistance against disease-causing pathogens. Two other plant insect resistance genes, *Mi-1.2* and *Vat*, have since been cloned; both likewise encode NB–LRR proteins [27], [28]. These three insect resistance genes originate from different plants but reveal similarities between the molecular mechanisms of insect and disease resistance. A receptor-like kinase gene *OsLecRK* was recently shown to be involved in basal defense response to brown planthopper attacks [29] and may be a PRR that recognizes molecules secreted by these insects.

Despite decades of investigation, the genetic basis of virulence in *N. lugens* remains to be identified. Genetic studies on this agriculturally important insect have been hindered by a lack of genome-wide linkage resources. However, it is known to exhibit considerable individual variation within biotypes [30]–[32]. Crossing experiments using selected biotypes indicate that the virulence is continuously distributed in the offsprings and cannot be predicted using simple Mendelian models. These results led to the conclusion that BPH virulence is subject to polygenic control rather than being monogenically inherited [33]–[35]. Nowadays, genomic tools can be used to dissect the genetic basis of *N. lugens* virulence. A large number of expression sequence tags (ESTs) have been generated and released by transcriptome analyses of different tissues of this insect [36]–[38], and polymorphic molecular markers from the *N. lugens* genome have been generated for various studies. The genetic diversity of different laboratory populations [30] and natural populations [39]–[41] has been assessed using molecular markers. Moreover, work towards a genetic linkage map for the brown planthopper has been initiated [42]. In this study, our objective was to reconstruct a high density genetic linkage map for the brown planthopper, and to identify the loci responsible for the virulence of Biotype 2 by using genome-wide linkage mapping. Our results revealed that the virulence of the planthopper biotype that overcame the first known rice resistance gene is controlled by major genes.

## Results

### Virulence of brown planthoppers on rice plants

**Figure 1:**
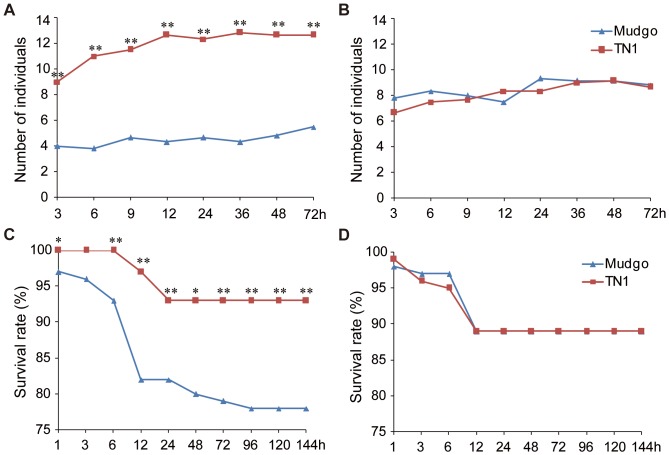
Host preferences and survival rates for Biotype 1 and 2 on TN1 and Mudgo rice plants. (A) and (B) The host preferences of Biotype 1 and 2 were assessed by recording the number of insects that settled on TN1 and Mudgo plants during 72h. Much more Biotype 1 insects chose the susceptible rice plants (TN1) than did resistant plants (Mudgo), while the Biotype 2 insects that settled on both rice plants exhibited similar number. (C) and (D) The survival rates of Biotype 1 and 2 insects were tested on TN1 and Mudgo plants by recording the number of nymphs. The Biotype 1 insects died much more individuals on resistant plants (Mudgo) than did on the susceptible rice plants after releasing 12 h, whereas the Biotype 2 insects survived well on both rice plants. *, Student's t-test, *P*<0.05; **, Student's t-test, *P*<0.01.

Two *N. lugens* biotypes were sib-mated for use in this work: Biotype 1 that had been reared on the rice variety TN1 (which carries no resistance gene), and Biotype 2 that had adapted to feed on the resistant Mudgo rice variety (which carries the *Bph1* resistance gene) (IRRI, 1976). Although we have previously detected genetic differences between the two populations [30], there were no obvious morphological differences between individuals of two biotypes. To characterize the virulence phenotypes of the two biotypes, the insects' biological responses to different rice plants were monitored, including their host preferences, level of nymph development, and growth rate [32]. Host preference was assessed by putting planthoppers in containers where they could choose between TN1 and Mudgo plants, and recording the number of insects that settled on each rice plant. As expected, the Biotype 1 insects exhibited a strong preference towards the susceptible TN1 variety and tended to settle on TN1 plants rather than the resistant Mudgo plants (Figure 1A). In contrast, the Biotype 2 insects had no strong preference for either rice variety (Figure 1B). Nymphal development was evaluated by placing newly emerged nymphs of each biotype onto either TN1 or Mudgo plants. Nymphal growth and mortality were recorded on a daily basis in each case until the nymphs either emerged as adults or died. The Biotype 1 insects exhibited much higher mortality rates on Mudgo plants than on TN1 plants whereas Biotype 2 insects exhibited similar mortality rates on both rice varieties (Figure 1C and D). Both biotypes had development times (i.e. the number of days required for half of the nymphs to emerge as adults) of 15 days on both host plants. Finally, the growth rate was calculated as the proportional difference in body weight between brachypterous females that had newly emerged on the test rice plants relative to their weight after feeding for 72 hours. The growth rates for the two biotypes on TN1 plants did not differ significantly (Table 1). However, the bodyweight of the Biotype 2 insects on the Mudgo plants increased to a greater degree than that of Biotype 1 insects (t-test, *P*<0.01). These results demonstrate that both Biotype 1 and Biotype 2 insects are virulent towards the susceptible TN1 rice variety, but only the Biotype 2 insects have adapted to and are virulent towards Mudgo plants that express the *Bph1* resistance gene.

**Table 1:** The mean performance of two *N. lugens* populations on TN1 and Mudgo plants.

Populations	Performance on TN1				Performance on Mudgo			
	*N*	Mean	SD	*P*-value	*N*	Mean	SD	*P*-value
Biotype 1	55	0.91	0.31	0.669	61	0.80	0.32	0.005
Biotype 2	53	0.88	0.28		60	0.95	0.26	

*N*, the number of individuals; Mean, the average proportional change in weight relative to initial weight; SD, the standard deviation; *P*-value, the value of *P* as determined using Student's t-test.

### Construction of a high density molecular map for brown planthopper

**Figure 2:**
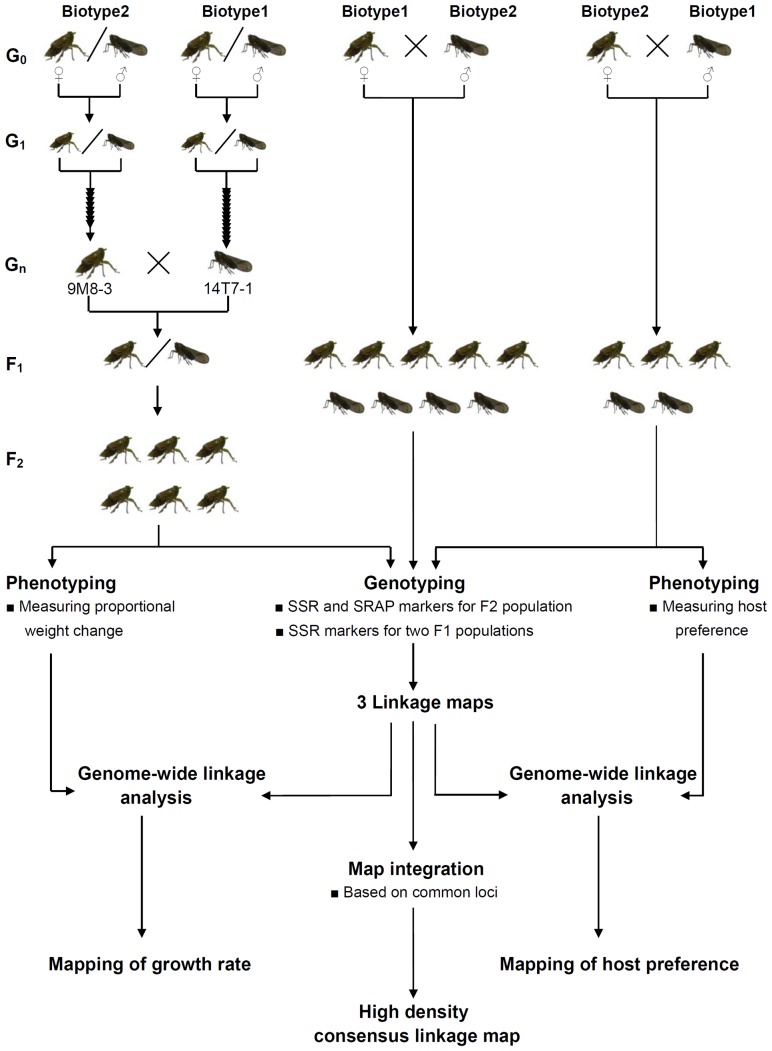
Schematic of the experimental design. Three mapping populations were used for constructing a high linkage map for brown planthopper and mapping virulence genes. The first one is termed as F_2_ population which crossed between 9M8-3 and 14T7-1. 9M8-3 is a virgin female individual from an inbred line of the Biotype 2 population generated by 9 generations of sib-mating; 14T7-1 is a virgin male individual from an inbred line of the Biotype 1 population generated by 14 generations of sib-mating. The other ones are two F_1_ populations (termed as F_1_(TM) and F_1_(MT)) which crossed between different individuals from Biotype 1 or Biotype 2. “Biotype 1” and “Biotype 2” refer to open-bred individuals from the corresponding populations.

Three brown planthopper populations were used for mapping in this work (Figure 2). The first population was termed the F_2_ population which is one of greatly efficient mapping populations [43]–[45] and consisted of 106 individual females that were generated by crossing a female of the inbred Biotype 2 line 9M8-3 and a male of the inbred Biotype 1 line 14T7-1. The other two mapping populations were termed F_1_ populations. The F_1_(TM) population consisted of 154 individuals arising from a cross between a Biotype 1 female and a Biotype 2 male, while the F_1_(MT) population consisted of 51 individuals arising from a cross between a Biotype 2 female and a Biotype 1 male. The grandparents of the F_2_ population were derived from individuals of inbred lines by sib-mating, but the parents of the two F_1_ populations were individuals from appropriate free-mating populations. It was anticipated that analyses of crosses between inbred individuals would enhance the scope for detecting genetic loci responsible for virulence, while analyses of open-mated individuals would provide a level of genetic variation that would enhance the linkage map's density.

In order to construct a high density linkage map of *N. lugens*, 833 EST-SSR primer pairs were newly developed from transcript sequences for this insect in public databases [37] and unpublished data generated within our group. These were combined with 366 recently released EST-SSRs [30], [42] and 745 genomic SSRs [39], [41], [42] to yield a total of 1944 SSR primer pairs that were used to characterize the parents of the three mapping populations. This set of primers was used to perform a preliminary PCR amplification screen for the parents of the F_2_ and two F_1_ populations. The numbers of the polymorphic markers in each case were 651, 434 and 405, respectively. After discarding markers that were un-scored due to their complex patterns of segregation or unclear amplification, 548 reliably scored markers were selected for linkage analysis in the F_2_ mapping population. The numbers of informative markers for the F_1_(TM) and F_1_(MT) mapping populations were 323 and 318, respectively. In total, 766 SSR markers were identified as being informative for linkage analysis. Details of the relevant primer sequences and the PCR product sizes for all of the SSR markers are presented in tables S1 and S2.

Sequence-related amplified polymorphism (SRAP) is a novel PCR-based molecular marker technique that was developed for the amplification of open reading frames [46]. The use of SRAP markers increases the density of linkage maps and also enables the identification of functional markers. In this work, 106 primer combinations including 25 forward and 36 reverse primers (Table S3) were used in the linkage analysis of the F_2_ population. 200 SRAP polymorphic loci were generated, giving an average of 1.89 polymorphic loci per primer combination.

In total, 966 informative SSR and SRAP markers were used to genotype individuals from the three mapping populations. A segregation analysis was performed, and those markers that were found to be distorted (*P*<0.0001) were excluded from subsequent analyses. This left 707 markers for use in the F_2_ mapping population, 302 in the F_1_(TM) population, and 305 in the F_1_(MT) population.

Linkage maps were constructed for each of three populations using the JoinMap 4.0 program [47]. The three linkage maps all had similar lengths: 867.0 cM for the F_2_ population (Table S4), 847.9 cM for the F_1_(TM) population (Table S5) and 779.8 cM for F_1_(MT) population (Table S6). The linkage maps for the F_2_ and F_1_(MT) populations consisted of 15 linkage groups while that for the F_1_(TM) population consisted of 14 linkage groups due to lack of segregation between X chromosome-specific markers. The average distance between loci in the maps ranged from 1.4 cM for the F_2_ population to 3.3 cM for the F_1_(MT) population.

**Figure 3:**
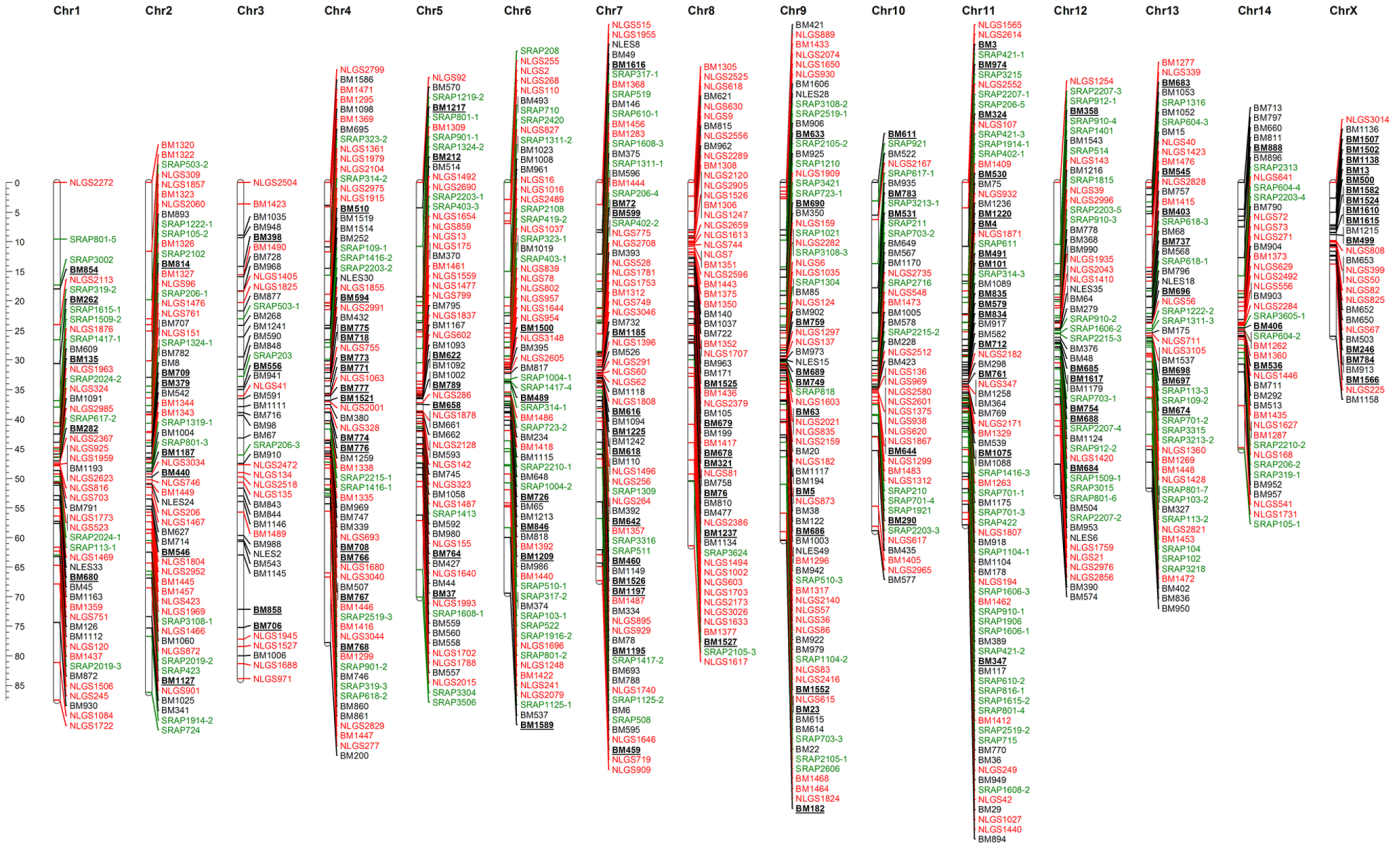
The high density molecular linkage map for the brown planthopper (*N.lugens*). The molecular markers with different colors (black for EST-SSR; red for genomic SSR; green for SRAP markers) didn't evenly distribute across each of 15 linkage groups and the characteristics of this map are summarized in Table 2. Markers whose names begin with NLES or NLGS are EST-SSRs or genomic SSRs from a previous map [42]. Those whose names begin with BM or SRAP are microsatellite and SRAP markers developed in our laboratory. The anchoring EST-SSR markers are indicated in bold text and underlined; detailed information on these markers is presented in Table S8. The number of these gene specific markers per chromosome ranged from 3 to 15, with an average of 8.

A consensus linkage map was constructed based on the loci that were common to all three or two populations (259 markers) by using the “combine groups for map integration” function of the JoinMap 4.0 program. The consensus molecular map contained 886 markers (706 microsatellites and 180 SRAPs) with 868 unique positions in 15 linkage groups, which matches the brown planthopper's haploid chromosome number. Based on their lengths in the consensus map, the linkage groups were designated Chr1-Chr14 (corresponding to the brown planthopper's 14 autosomes) and ChrX corresponding to the X sex chromosome. The characteristics of the consensus map are summarized in Table 2, and the distributions and positions of the markers across linkage groups are shown in Figure 3 and Table S7.

**Table 2:** Overview of the consensus molecular linkage map for the brown planthopper.

aLG	Length (cM)	No. of loci	No. of marker	Average distance (cM)	Genome coverage (%)	Largest interval (cM)	EST-SSR	bG-SSR	SRAP	Jairin's map
Chr1	87.5	48	50	1.82	96.1	9.6	16	23	11	4
Chr2	86.2	60	60	1.44	96.7	11.6	19	28	13	8
Chr3	83.9	46	46	1.82	95.7	9.1	28	15	3	15 and 17
Chr4	77.8	70	70	1.11	97.2	11.1	32	27	11	5
Chr5	70.1	63	64	1.11	96.9	8.5	28	26	10	1
Chr6	69.4	69	69	1.01	97.1	9.4	22	26	21	9 and 16
Chr7	67.3	73	76	0.92	97.4	5.8	33	30	13	7
Chr8	61.4	58	61	1.06	96.8	10.9	21	38	2	6
Chr9	60.5	77	80	0.79	97.5	5.3	34	31	15	2
Chr10	58.9	45	46	1.31	95.7	9.4	16	19	11	10
Chr11	57.9	82	83	0.71	97.6	6.5	37	20	26	3
Chr12	52.9	52	53	1.02	96.3	10.5	23	12	18	13
Chr13	51.7	55	56	0.94	96.5	5.5	22	17	17	12
Chr14	45.1	43	43	1.05	95.5	7.1	16	18	9	14
ChrX	26.0	27	29	0.96	93.3	12.2	21	8	0	11
Total	956.6	868	886	1.10	96.6		368	338	180	

a LG, linkage group of consensus molecular linkage map;

b G-SSR, the genomic SSR;

Jairin's map, the linkage groups according to a previous map [42].

In summary, this molecular linkage map of brown planthopper spanned 956.6 cM in total, covering 96.6% of the species' estimated genome size. The lengths of individual linkage groups ranged from 26.0 cM (ChrX) for the shortest to 87.5 cM (Chr1) for the longest. The linkage group with the greatest marker density was Chr11, with an average distance between markers of 0.71 cM. Those with the lowest marker densities were Chr1 and Chr3, for which the average distance between markers was 1.82 cM. Across the whole map, there were only 6 cases in which the distance between adjacent markers was greater than 10 cM; the largest gap was on ChrX and extended over 12.2 cM. The number of markers per linkage group ranged from 29 (ChrX) to 83 (Chr11), with an average of 59 markers per group. The new consensus map thus provides greatly enhanced marker density and resolution compared to that available previously.

The use of gene-based markers made it possible to map genes with known functions onto the new linkage map. Public databases were searched to identify genes and proteins of known function corresponding to the 322 EST sequences and the 357 associated EST-SSRs. No BLAST hits could be identified for the shorter EST sequences. However, 110 EST sequences corresponding to 124 EST-SSRs were matched to proteins or genes of known function with E values of <10^−5^ (Table S8). These gene-specific SSRs were used as anchor markers for each chromosome (Figure 3). The number of gene specific markers per chromosome ranged from 3 to 15, with an average of 8. The linkage group Chr1 had five gene-specific EST-SSRs, two of which were matched to the brown planthopper genes encoding the neuropeptides precursor and angiotensin converting protein (ACE). Genes encoding reverse transcriptase, calcium calmodulin-dependent protein kinase, replication protein and pre-mRNA-splicing factor were mapped to the Chr2 linkage group. These genes are important for essential cellular functions. The genes mapped to the Chr3 linkage group included those encoding guanine nucleotide exchange factor and cell division cycle-associated protein. Six different EST-SSRs associated with the gene encoding methionine-R-sulfoxide reductase were identified in the Chr4 linkage group. While some of these SSRs were located in close proximity to one-another, others were more widely separated. This may reflect the presence of large introns in this gene. A gene encoding anacetylcholinesterase (*ace-2*) was also mapped to this chromosome. Markers for the acetylcholinesterase (*ace-1*) gene and genes encoding zinc finger proteins were mapped to the Chr5 linkage group. Linkage group Chr6 was associated with genes encoding microtubule-associated protein and pyridoxine pyridoxamine 5-phosphate oxidase. The brown planthopper genes encoding neuropeptide GPCR A5 and cytochrome P450 CYP6ER1 were mapped to linkage group Chr7, along with markers associated with EBNA2 binding protein p100 and ribosomal proteins. Linkage group Chr8 featured markers associated with the mucin-like protein gene, which may be important for the feeding behavior of brown planthoppers. Markers associated with histone RNA hairpin-binding protein, silencing protein and cysteine proteinase inhibitor precursor were mapped to the Chr9 linkage group. The Chr10 linkage group contained two markers for cytochrome P450 CYP6ER1, while the Chr11 linkage group contained 15 gene-specific annotated SSRs corresponding to the actin and chitin deacetylase genes. The EST-SSRs of the Chr12 linkage group were associated with chromodomain-helicase-DNA-binding protein and angiotensin converting protein (ACE). The Chr13 linkage group contained three EST-SSRs corresponding to the tyrosine-protein phosphatase gene. Only 3 gene-specific SSRs were anchored in the linkage group Chr14, corresponding to the gene for ESF1-like protein. Finally, 10 EST-SSRs corresponding to the vitellogenin gene were identified in the ChrX linkage group, along with markers for the transposase-like protein and death-associated protein genes.

### Mapping for virulence genes

Plants have three mechanisms by which they can resist insect attacks: antixenosis, antibiosis and tolerance. Antixenosis and antibiosis refer to the response of insect to plant, mainly as reduced colonization, survival, growth rate, or reproduction. The capacity of brown planthoppers to overcome antixenosis and antibiosis responses in rice can thus be evaluated by studying their host preferences and growth rates.

The F_1_ population generated from the Biotype 2/Biotype 1 cross was used to dissect the genetic basis for host preference. The virulence phenotype of the F_1_ progeny with respect to host preference was assessed by counting the number of nymphs that settled on TN1 plants or Mudgo plants when given a choice. We found that more individuals settled on the Mudgo plants (43 individuals) than on the TN1 plants (8 individuals) in the F_1_ population. Because the Mudgo variety expresses the resistance gene *Bph1*, this preference for Mudgo plants indicates that the virulence of the F_1_ population is sufficient to overcome the virulence of *Bph1*. This finding also suggests that virulence is a dominant trait.

**Figure 4:**
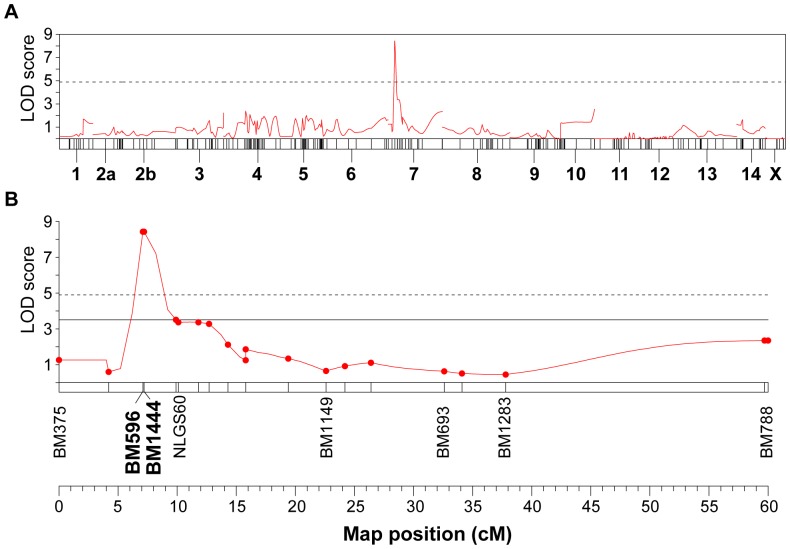
Mapping of loci relating to host preference in brown planthopper. (A) The results of a genome-wide QTL scan for host preference on the linkage map for the F_1_ population. A QTL reached significantly on chromosome 7 (7.1 cM). Host preferences were evaluated as a binary trait (“1” is settled on TN1 rice plants, “2” is settled on Mudgo rice plants). The horizontal bars represent 15 linkage groups. (B) Location and effect of the host preference gene *Qhp7* on chromosome 7. It was located on a small region of chromosome 7 (between markers BM596 and BM1444, in an interval that is 0.1cM in length) and explained 54.0% of phenotypic variation from F_1_ population. The dashed horizontal line indicates the significant genome-wide threshold LOD score (LOD = 4.9); the solid line indicates the significant linkage group-wide threshold LOD score (LOD = 3.5).

To identify the genetic loci responsible for this virulence, we analyzed the host preference phenotype data in conjunction with the molecular marker data for the F_1_ population using MapQTL 6.0 [48]. A genome-wide linkage scan identified one locus that affected host preference (Figure 4A). A QTL analysis for virulence was performed using interval mapping (IM) with 5% genome-wide and linkage group-wide thresholds, which were calculated from 10000 permutation iterations of the quantitative trait data. A significant locus for host preference was thus identified in the F_1_ population (Figure 4B). This locus was mapped on chromosome 7 between molecular markers BM596 and BM1444, in an interval that is 0.1 cM in length. The LOD score for the locus was 8.43, which is much higher than the genome-wide threshold of 4.9. It explained 54.0% of the phenotypic variation (PVE) in the population and was named *Qhp7* (Table 3).

**Table 3:** Quantitative trait loci identified for virulence traits in the F_1_ and F_2_ populations.

Traits	QTL	LG	Interval	Distance (cM)	Position (cM)	A	D	LOD	PVE (%)
Host preference	*Qhp7*	F_1_(MT)-7	BM596-BM1444	0.1	7.1			8.43^a^	54.0
Growth rate	*Qgr5*	F_2_-5	NLGS859-NLGS13	0.3	34.1	0.188	-0.059	3.91^b^	17.8
	*Qgr14*	F_2_-14	NLGS72-SRAP604-4	1.1	14.5	0.172	-0.165	5.48^c^	24.0

LG: Linkage group for the F_1_ or F_2_ population; A: Additive effect; D: Dominance effect;

PVE: percentage of the phenotypic variance explained in mapping population;

a:The Genome-wide and Linkage-group-wide LOD thresholds are 4.9 and 3.5, respectively;

b:The Genome-wide and Linkage-group-wide LOD thresholds are 4.5 and 3.1, respectively;

c:The Genome-wide and Linkage-group-wide LOD thresholds are 4.5 and 2.9, respectively.

**Figure 5:**
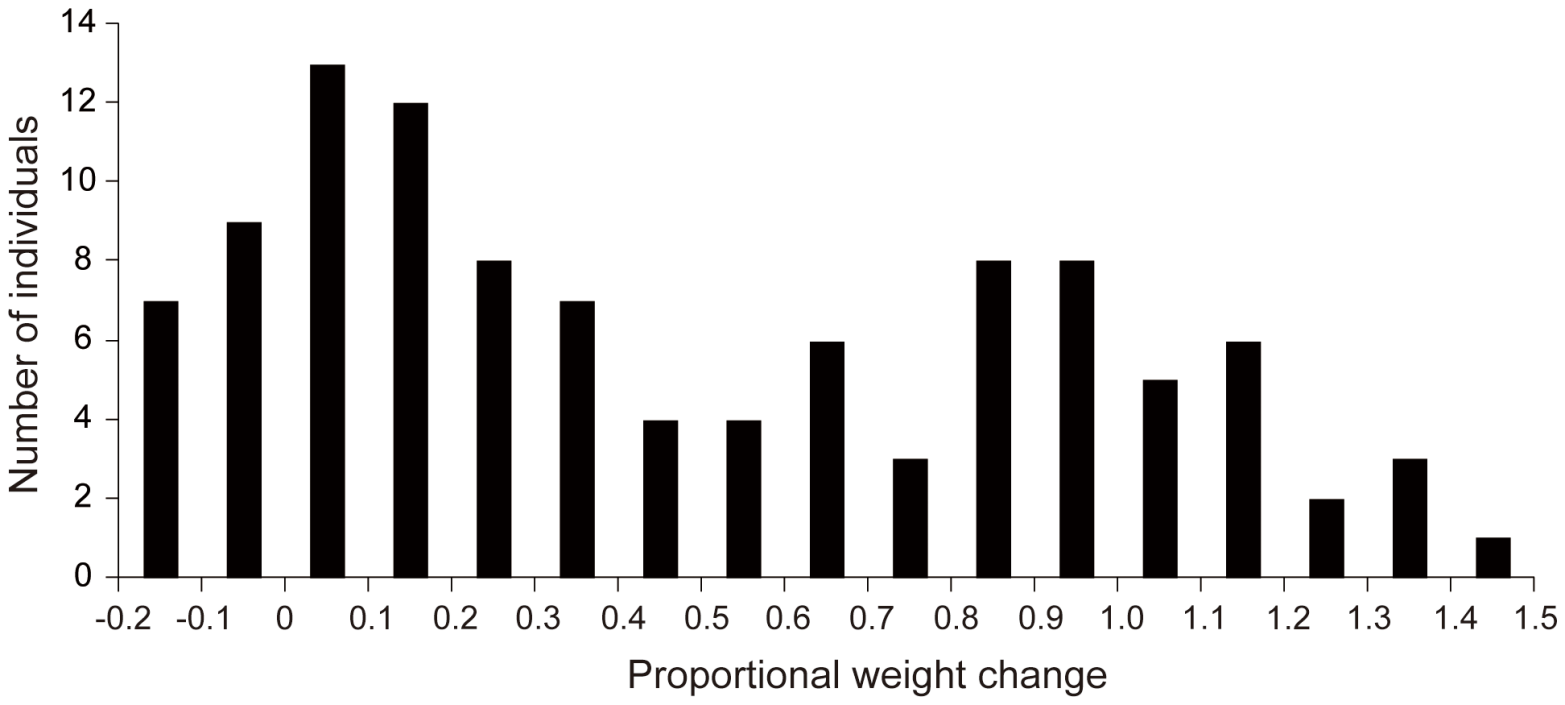
Frequency distribution of growth rates for brown planthopper females from the F_2_ population on Mudgo plants.

The F_2_ population derived from the Biotype 2/Biotype 1 cross was used to study the genetic factors responsible for the variation in the insects' growth rate. The virulence phenotype of the F_2_ progeny with respect to growth rate was assessed by measuring the proportional change in weight of females on the resistant Mudgo plants between the time when they were first deposited on the plants and a point 72 hours later. The observed changes in weight for the F_2_ individuals ranged from a low of -0.18 to a maximum of 1.44, with an average value of 0.47. The distribution of phenotypic scores among the F_2_ progeny (n = 106) was bimodal (Figure 5), indicating that major genes control the virulence that enables some individuals in this population to overcome the antibiosis conferred by *Bph1*.

**Figure 6:**
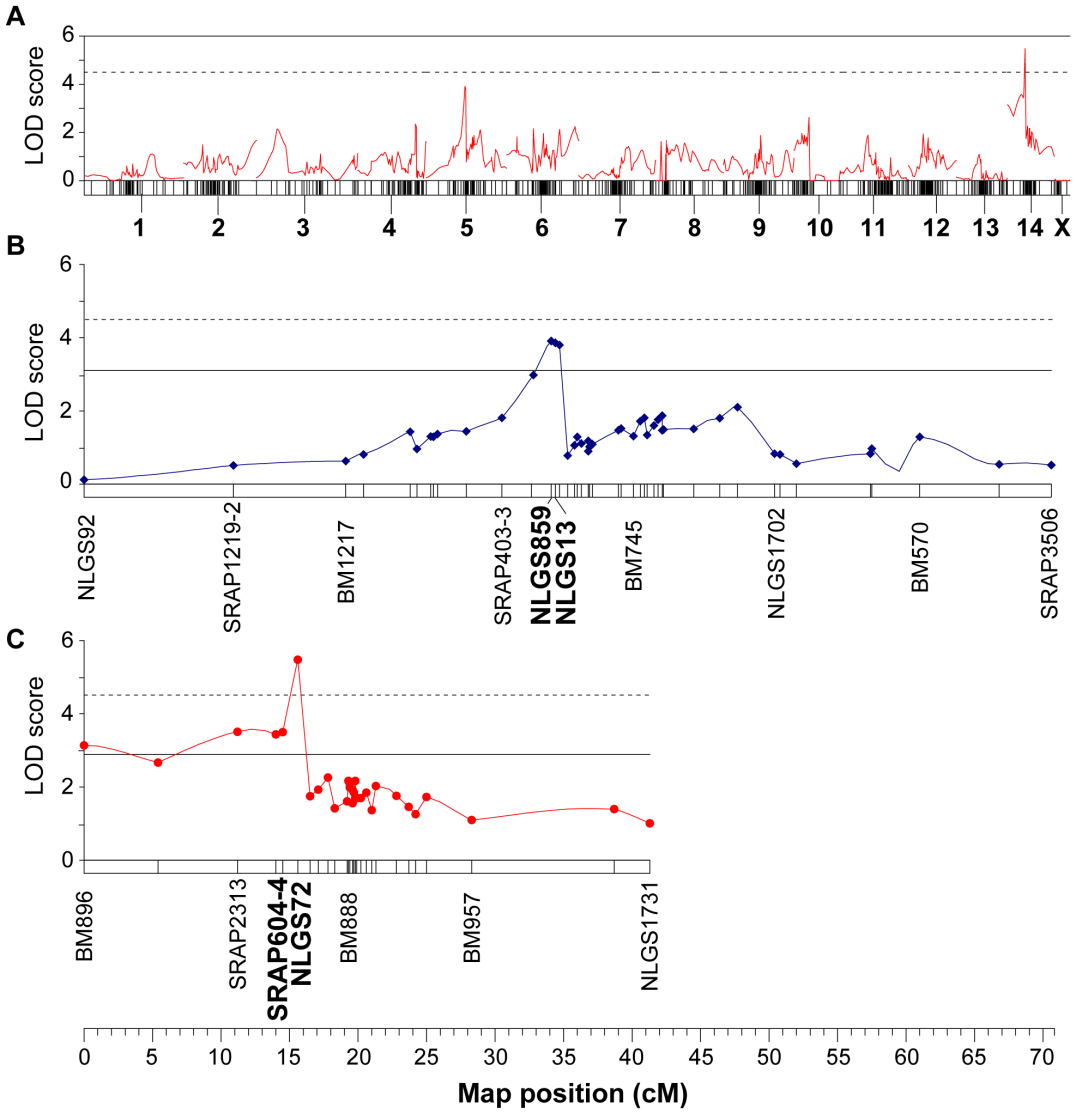
Mapping of loci for growth rate among brown planthoppers on resistant rice plants. (A) Genome wide QTL scan for growth rate on the linkage map for the F_2_ population. Two QTLs reached significant on chromosome 5 (34.1 cM) and 14 (14.5 cM), respectively. The horizontal bars represent 15 linkage groups. (B) Location and effect of the growth rate gene *Qgr5* on chromosome 5. It was located on a small region of chromosome 5 (between markers NLGS859 and NLGS13, their interval distance is 0.3 cM) and explained 17.8% of phenotypic variation from F_2_ population. (C) Location and effect of the growth rate gene *Qgr14* on chromosome 14. It was located on a region of chromosome 14 (between markers NLGS72 and SRAP604-4, their interval distance is 1.1 cM) and explained 24.0% of phenotypic variation in F_2_ population. The dashed horizontal line indicates the significant genome-wide threshold LOD scores (LOD = 4.5); the solid line indicates the significant linkage group-wide threshold LOD scores (LOD = 3.1 for chromosome 5 and LOD = 2.9 for chromosome 14).

To identify genetic loci that control the growth rate, a genome-wide linkage scan was conducted using phenotype and molecular marker data for the F_2_ population. Two QTLs associated with the growth rate were detected (Figure 6A). Further analysis using interval mapping revealed that a QTL with a LOD score of 3.91 was located on chromosome 5 between markers NLGS859 and NLGS13 (Figure 6B). The interval distance for this QTL was 0.3 cM and it accounted for 17.8% of the population's phenotypic variation (Table 3). This locus was named *Qgr5*. Another growth rate-associated QTL was mapped on chromosome 14 (Figure 6C), with a LOD score of 5.48. It was located between the NLGS72 and SRAP604-4 markers, in an interval of 1.1 cM. This QTL explained 24.0% of the phenotypic variation in the population and was named as *Qgr14* (Table 3). Both QTLs exhibit additive and dominant effects, and no significant interaction was detected between them.

## Discussion

We have constructed the most comprehensive linkage map for *N. lugens* that is currently available and demonstrated that brown planthopper virulence towards rice plants is controlled by a small number of genetic loci. To the best of our knowledge, this is the first study to successfully locate virulence factors in the genome of this important agricultural insect by marker-based genetic mapping. Building on the previous results [42], we constructed a high density molecular linkage map for the brown planthopper genome in order to enable the mapping of specific genes. The loci governing host preference and growth rate were delimited to specific regions flanked by molecular markers. This represents a critical first step towards identifying the mechanisms that underpin insects' virulence and ability to overcome host plant resistance.

High-resolution genetic linkage maps are becoming increasingly important in genetic and genomic studies [49]. In this study, several methods were used to construct a high-resolution linkage map for the brown planthopper. In addition to previously identified markers, a number of new SSRs mined from transcriptome databases within our group were developed and SRAP markers were also used for the first time in *N. lugens*. Overall, 2144 unique markers were considered in this work, of which 966 were informative for map development. The information provided by these molecular markers represents a valuable resource for genetic and genomic studies on this insect. Three populations were analyzed in the construction of the map. Brown planthoppers are known to exhibit high levels of heterozygosity [30], [50], and the number of successful progeny per mating is limited. We therefore constructed a consensus linkage map by integrating information from multiple families. Comparing to previous genetic map of this insect [42], the basic characteristics was significantly improved in this map. First, it consists of 15 linkage groups that coincide with the species' 14 autosomes and one X chromosome. Conversely, the map developed by Jairin *et al.* features 17 linkage groups. In total, 283 of the markers used in Jairin's map were incorporated into our consensus map. As a result, we were able to combine LG9 and LG16 from the Jairin's map into a single group corresponding to chromosome 8, and to combine the Jairin's linkage groups LG15 and LG17 into a group corresponding to chromosome 12. Unfortunately, additional markers will be required to construct a linkage map for the Y chromosome. According to their length in cM, to these 15 linkage groups, which should greatly facilitate studies hereafter. We numbered the linkage groups based on their lengths in cM, which should greatly facilitate future studies using our map. The map spans 956.6 cM, which corresponds to 96.6% of the estimated size of the *N. lugens* genome. It is based on 886 molecular markers with an average distance between adjacent markers of 1.1 cM. Such high density maps covering the whole genome of the target species are valuable tools for dissecting the genetic basis of important traits. Another prominent merit of our molecular map is the use of gene-specific markers as anchors on each chromosome. On average, there are eight anchor markers per chromosome. The sequences from which the markers were developed are over several hundred bases in length and are known to correspond to a specific protein or gene. These markers function as chromosomal landmarks, especially when they cluster together. For example, the vitellogenin gene on the X chromosome was previously located on the sex linkage group in previous maps [42]. However, based on our map, we were able to demonstrate that the transposase-like protein and death-associated protein 1 are also located on this chromosome. The chromosomes of the brown planthopper have been observed, but the cytological karyotype of this species has not yet been established. The gene-specific anchor markers identified in this work will provide an opportunity to integrate our molecular linkage map with cytological karyotype data. It will then be possible to exploit genomic in situ hybridization techniques to locate gene-specific anchor markers for individual linkage groups on the corresponding chromosome.

**Figure 7:**
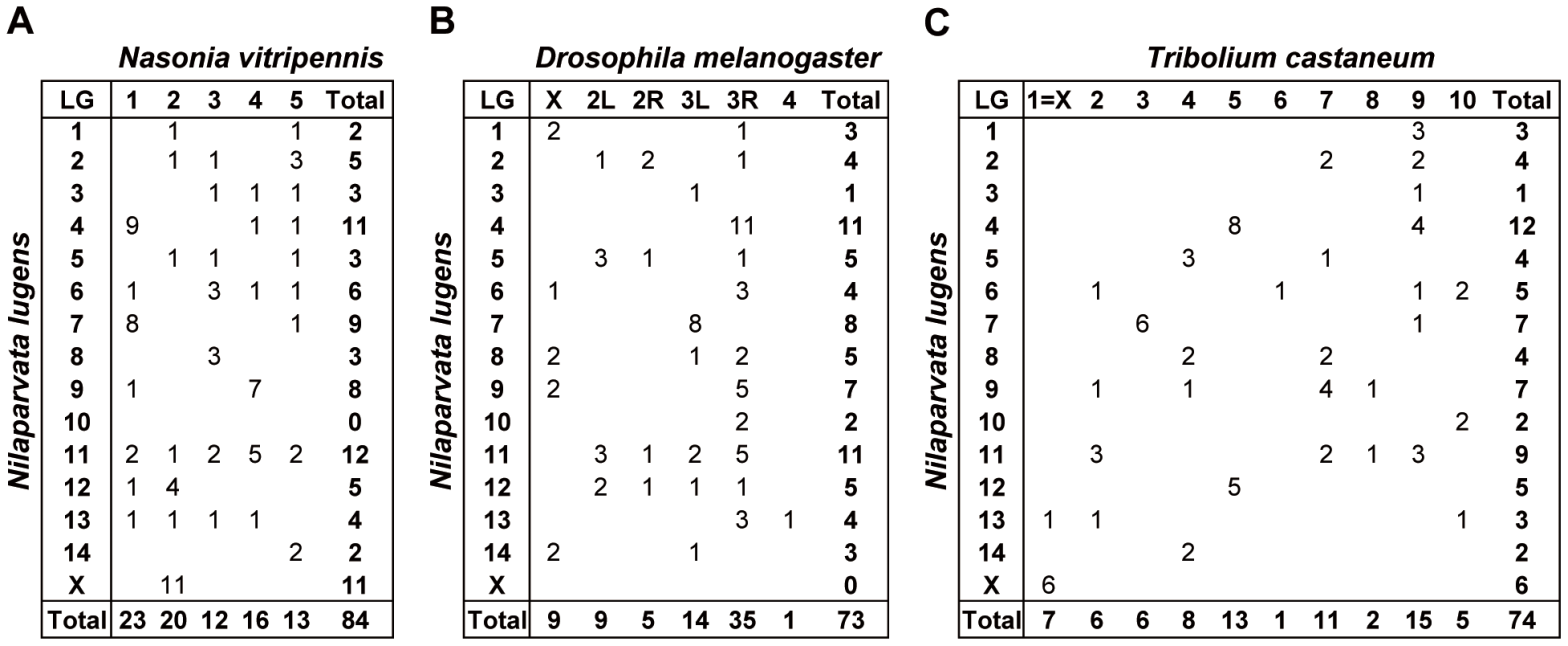
Oxford grids displaying a matrix of cells comparing the number of orthologous genes on chromosomes of *N. lugens* and selected model insects. (A) Much more orthologous genes were found between *Nilaparvata lugens* and *Nasonia vitripennis* comparison, indicating that a high syntenic relationship between these two insects. (B) and (C) The number of orthologous genes between *Nilaparvata lugens* and *Drosophila melanogaster* comparison and that of *Nilaparvata lugens* and *Tribolium castaneum* comparison were nearly same, indicating that their syntenic relationship with brown planthopper was similar.

The sequences of gene-specific markers also provide a basis for comparing the relationships between different species' genomes [51], [52]. Specifically, they can be compared to those for genes in a model genome to identify orthologs, and their chromosomal positions can be used to define the extent of conservation between brown planthopper and the chosen model species. To illustrate this process, 110 *N. lugens* EST sequences were searched against complete genome data for four other insect species. In total, 90 orthologs were found in the *Acyrthosiphon pisum* genome, 88 in *Nasonia vitripennis*, 83 in *Tribolium castaneum*, and 76 in *Drosophila melanogaster*. Homology segments were identified and there was a strong syntenic relationship between the brown planthopper and the model insects (Figure 7). The addition of further EST markers to the map will enable more comprehensive comparative genomics analyses of the relationships between brown planthoppers and other insects.

Much progress has been achieved in understanding the genetic and molecular mechanisms that enable rice to resist brown planthopper attacks [11]. It is believed that major genes control resistance while minor genes or QTLs contribute to the durability of the resistance mechanisms. Brown planthopper feeding activates resistance genes, which in turn triggers the plant's defense reactions. Signal transduction then causes the reprogramming of the transcriptome and proteome, inducing resistance mechanisms such as sieve tube blocking and the production specific materials that deter planthoppers. Processes of this sort underpin resistance based on antixenosis and antibiosis.

When a planthopper feeds on rice plants, it begins by settling on an appropriate plant and then uses its stylet to penetrate the plant's cell walls. The insect then salivates into the cells and ingests the phloem sap of plant. To overcome the plant's resistance mechanisms and preserve its virulence, the insect must withstand both antixenosis and antibiosis. Previous studies suggested a model of polygenic inheritance for virulence genes in brown planthoppers [35], [53]. When studying the genetics of virulence, it is essential to use appropriate phenotype scores. In this work, we targeted host preference, i.e. the number of insects that settled on rice plants expressing the *Bph1* resistance gene compared to susceptible TN1 plants, as the virulence phenotype that breaks antixenosis. Two phenotypes were possible, reflected by the individual insect's decision to settle on either a TN1 or a Mudgo plant. Genome-wide scans revealed a locus that had significant effects on this phenotype, *Qhp7*. This gene was located on chromosome 7 between markers BM596 and BM1444. Its identification demonstrates that one aspect of virulence, i.e. the insect's preference for resistant rice varieties expressing *Bph1*, is largely determined by a major gene.

To identify virulence factors that countered the antibiosis effect of *Bph1*, we studied the growth rate of insects feeding on resistant plants rather than honeydew production. In our experience, body weight changes provide more accurate and direct information than honeydew-based approaches. The growth rate varied continuously in the F_2_ population when it was only allowed to feed on Mudgo plants, but the distribution was clearly bimodal, suggesting monogenic or oligogenic inheritance. Two major QTLs, *Qgr5* and *Qgr14*, were mapped to very narrow segments of chromosome 5 and 14, demonstrating that the virulence that overcame the antibiosis effect of *Bph1* was controlled by an oligogenic system.

These results advance our understanding of the molecular genetic basis for of virulence in of the *N. lugens*. In general, insect virulence is governed by components that match the mechanisms of host resistance. In the case of the brown planthopper and rice, the virulence factors affecting host preference act against antixenosis, while those affecting growth rate act against antibiosis. The components of insect virulence might derive from independent genetic characters. The practical implication of this for rice improvement is that the integration of multiple resistance mechanisms into new varieties will delay the formation of new herbivorous insect biotypes. Because resistance to brown planthopper feeding in rice varieties is known to be controlled by major genes [24], [26], it is widely assumed that there is a gene-for-gene relationship between resistance on the part of the rice plant and virulence on the part of the pest. It will be interesting to carry out fine-scale mapping experiments to isolate the genes responsible for host preference and growth rate, and to identify allelic variations related to virulence: this will shed light on the coevolutionary interactions between plant and insect, and allow us to better understand how to delay or prevent the adaptation of this pest to the available resistance genes in long term. The very comprehensive *N. lugens* linkage map presented in this work will thus provide new insights into the evolution of planthopper genomes and will be a valuable tool for validating genome sequence assemblies, detecting QTLs, map-based cloning, assessments of genetic diversity, and comparative genomic studies.

## Materials and Methods

### Rice plants

Two rice varieties were considered. The TN1 variety expresses no resistance gene and is susceptible to brown planthoppers of Biotypes 1 and 2. The Mudgo variety expresses the *Bph1* resistance gene and is resistant to Biotype 1 planthoppers but not to those of Biotype 2 [31].

### Brown planthopper families

Brown planthopper insects of Biotype 1 and Biotype 2 were maintained on rice variety TN1 and Mudgo plants, respectively. In order to reduce the genetic variation in the populations, single pair sib-matings were conducted between successive generations of both biotypes (Figure 2). In addition, cross matings between insects of Biotype 2 and Biotype 1 were conducted. The resulting offspring were raised and tested in a greenhouse with a constant room temperature (25°C) at the Genetics Institute, Wuhan University.

### Phenotype investigation

To investigate the difference between the two brown planthopper biotypes, we characterized their biological reactions to susceptible and resistant rice plants. Host preference tests were conducted on four-week-old TN1 and Mudgo plants that were grown side by side in a cup (20 cm in diameter). Twenty nymphs of each biotype were place in the middle of the cup at the beginning of the experiment. The number of insects on each rice plant was then counted and recorded 3 h, 6 h, 9 h, 12 h, 24, 36 h, 48 h and 72 h after the start of the experiment. The experiment was repeated six times.

To investigate nymphal development, twenty newly emerged first instar nymphs of each biotype were placed on four-week-old TN1 or Mudgo plants. The mortality of nymph was recorded at 1 h, 3 h, 6 h, 12 h, 24 h, 48 h, 72 h, 96 h, 120 h and 144 h. Moreover, the day on which half of the nymphs had emerged as adults was noted. This experiment was repeated five times.

Growth rates were studied using four-week-old rice plants. Newly emerged brachypterous females were weighed within three hours of emergence on a microbalance to obtain an initial weight. The insects were then placed on Mudgo or TN1 plant. After 72 h, each individual was weighed again. The growth rate was then calculated as the proportional change in weight relative to the initial weight. Four combinations of host variety and planthopper biotype were evaluated, and at least 50 individual insects were weighed for each combination (Table 1).

The virulence of the individuals in the mapping populations was assessed with respect to host preference for TN1 and Mudgo plants and with respect to growth rate on Mudgo plants.

All of the resulting data were analyzed using the statistical program SPSS 14.0 [54].

### Development of new molecular markers

#### Microsatellites (SSR)

New microsatellite markers were developed from sequences in EST databases as described elsewhere [30]. These markers were named according to our laboratory's convention, in which the first two letters (“BM”) reflect the name of the originating insect species (Brown planthopper Marker,).

#### Sequence-related amplified polymorphisms (SRAP)

Two-primer amplification was performed according to the SRAP protocol of Li and Quiros [46]. In this technique, both primers consist of core sequences which are 14 (forward primer) or 15 (reverse primer) nucleotides long and three selective bases at the 3′ end. The forward and reverse primers usually bind to exon and intron regions, respectively. Due to the variation of these regions among different individuals, polymorphic fragments are easily generated.

An SRAP profile can produce both co-dominant and dominant fragments. The presence/absence of bands for each marker was converted into the appropriate genotypes. SRAP markers were named using the prefix “SRAP” and a combination of numbers that denotes the reverse and forward primers. If a primer combination detected multiple loci, a suffix (-1, -2 and -3 etc.) was assigned to these loci names in order of descending fragment size. For example, SRAP1222-2 is the shorter of two fragments generated by the primer combination Em12 and Me22, while SRAP210 is the lone polymorphic fragment generated by the primer combination Em2 and Me10.

### Genotyping

The CTAB method [55] was used to extract DNA from individuals belonging to the mapping populations. The quality of the DNA was checked using agarose gel electrophoresis, and its concentration was measured using a Nanodrop instrument (NanoDrop, Wilmington, DE).

All PCR amplifications were performed using a PTC-100 thermal cycler (MJ Research) and 10 µl reaction mixtures containing 10 ng of template DNA, 0.3 µM of each of the two primers, 0.2 mM deoxynucleotide triphosphates (dNTPs), 2.5 mMMgCl_2_, 1×PCR buffer, and 1 unit of *Taq* DNA polymerase (Fermentas). The PCR cycling program for the SSR markers involved heating at 94°C for 5 min followed by 40 cycles of 94°C for 15 s, 55°C for 15 s, and 72°C for 30 s, with a final extension step of 72°C for 10 min. The PCR program for the SRAP markers involved an initial five cycles of 94°C for 60s, 33°C for 60s, and 72°C for 90s. This was followed by a further 35 cycles using an annealing temperature of 53°C. Genotypes were detected on 6% urea-denaturing polyacrylamide sequencing gels that were run at a constant power of 60 W. PCR amplification products were detected by silver staining [56]. Allele sizes were determined by comparing the products to pBR322 DNA/*Msp* I DNA size markers (Tiangen Biotech). The genotype of each marker was manually scored twice to reduce error.

### Linkage analysis

The JoinMap 4.0 program was used for linkage analysis and to construct maps for each mapping population [47]. According to the cross-pollinated (CP) population coding scheme of JoinMap 4.0, all markers were divided into four segregation types with null-alleles allowed: the 1∶1∶1∶1 type (female × male: ab× cd or ef × eg), the 1∶2∶1 type (hk × hk), the 1∶1 female type (lm × ll), and the 1∶1 male type (nn × np). These markers were then tested for goodness of fit to expected Mendelian segregation ratios using chi-square tests, and very significantly distorted markers (*P*<0.0001) were discarded from the data set to avoid creating spurious groups. The markers for each population were initially grouped with a LOD threshold 5.0. Once a framework map with 15 linkage groups (corresponding to the known haploid chromosome number of the brown planthopper) had been established, the remaining markers were added to the appropriate groups by setting a less stringent LOD threshold of 3.0. The regression mapping algorithm with the default settings (recombination frequency threshold <0.4, LOD threshold >1) was used to order loci within each linkage group. Then the Kosambi mapping function [57] was used to convert recombination frequencies into map distances. At first, markers were grouped for the F_1_ and F_2_ mapping populations. Based on data for the homologue linkage groups and the segregation data for each mapping population, a consensus map was constructed by performing an integrated linkage analysis using JoinMap 4.0. Graphic illustrations of the linkage groups were generated using MapChart 2.2 software [58].

The length of the genome was estimated in two ways from the consensus linkage maps. First, the average spacing between markers (*s*) was calculated by dividing the total length of all linkage groups by the number of intervals (number of markers minus number of linkage groups). The expected genome length (*Ge_1_*) was estimated by adding 2*s* to the length of each linkage group to account for the terminal regions of the chromosomes [59]. Second, according to the method of Chakravarti *et al.* [60], the expected genome length (*Ge_2_*) was estimated by multiplying the length of each linkage group by the factor (*m* +1)/(*m* - 1), where *m* is the number of markers on that linkage group. The coverage of consensus linkage maps was calculated as *Go*/*Ge*, where *Go* is the observed linkage group length and *Ge* is the average value of *Ge*_1_ and *Ge*_2_ for each linkage group [61].

### Comparative genome analysis

The sequences of the ESTs containing the microsatellites on linkage map were used in Blast2go (v2.7.0) searches against the Non-Redundant Protein Database to match known proteins using default parameters. These EST sequences were also used in BLASTN searches of the NCBI web database (http://blast.ncbi.nlm.nih.gov/Blast) to identify corresponding transcript sequences for brown planthopper genes. The synteny between brown planthopper and *Acyrthosiphon pisum, Drosophila melanogaster, Tribolium castaneum* or *Nasonia vitripennis* was evaluated based on sequence comparisons between brown planthopper ESTs harboring a mapped microsatellite and reference genome sequences. Finally, homologous orthologs of brown planthopper genes were identified by searching the appropriate chromosomal locations in the selected reference genomes using the NCBI Map Viewer (http://www.ncbi.nlm.nih.gov/projects/mapview).

### Identification of QTLs

QTL analysis was performed using the MapQTL 6.0 software package [48]. The selected virulence traits were host preference and growth rate. The host preference of the F_1_ population was recorded as “1”on TN1 plants or “2”on Mudgo plants while the growth rate of the F_2_ population was measured as described above. The interval mapping method [62] was used to identify significant associations between phenotypic variance and segregating genetic loci. LOD profiles along linkage groups with an interval distance of 1 cM were then generated to search for QTL locations within specific linkage groups. The genome-wide LOD threshold and individual LOD threshold per linkage group were calculated from permutation tests conducted with the MapQTL program using 10000 permutation iterations at a *P* value of 0.05 [63]. Once the LOD score of a linkage group exceeded the linkage group-wide LOD threshold (suggestive level) or genome-wide LOD threshold (significance level), a QTL was considered to have been detected. The percentage of phenotypic variance explained (PVE) for each locus was then calculated in MapQTL 6.0 based on the variance observed in the mapping populations. Graphic illustrations of the linkage groups and QTL profiles were generated using MapChart 2.2 [58].

## Supporting Information

Table S1 **List of 833 new EST-SSR markers developed in this work.**
(XLSX)

Table S2 **List of polymorphic genomic-SSR markers** [39], [41] **found in the parents of the F_1_ and F_2_ mapping populations.**
(XLSX)

Table S3 **The primers for the SRAP markers used in this study.**
(XLSX)

Table S4 **Summary of the molecular linkage map constructed for the F_2_ population.**
(XLSX)

Table S5 **Summary of the molecular linkage map constructed for the F_1_(TM) population.**
(XLSX)

Table S6 **Summary of the molecular linkage map constructed for the F_1_(MT) population.**
(XLSX)

Table S7 **Location of markers on the consensus map.**
(XLSX)

Table S8 **Detailed information on the 124 anchoring EST-SSR markers.**
(XLSX)
